# What public health interventions do people in Canada prefer to fund? A discrete choice experiment

**DOI:** 10.1186/s12889-022-13539-5

**Published:** 2022-06-13

**Authors:** Kiffer G. Card, Marina Adshade, Robert S. Hogg, Jody Jollimore, Nathan J. Lachowsky

**Affiliations:** 1 Faculty of Health Sciences, Simon Fraser University, Burnaby, BC Canada; 2 School of Public Health and Social Policy, University of Victoria, Victoria, BC Canada; 3 British Columbia Centre for Excellence in HIV/AIDS, Vancouver, BC Canada; 4 Community-based Research Centre, Vancouver, BC Canada; 5 Vancouver School of Economics, University of British Columbia, Vancouver, BC Canada

**Keywords:** Public Health, Interventions, Marginalized Communities, Public Opinion, Discrete Choice Experiments

## Abstract

**Objective:**

To assess public support of tailored and targeted public health interventions for marginalized communities.

**Methods:**

We conducted a discrete choice experiment using a web-based survey advertised to Facebook and Instagram users living in Canada, aged > 16. Participants were asked to choose between funding two hypothetical public health programs. Each program was described by its purpose; expected increase in life expectancy; and target audience. Demographically weighted generalized linear mixed-effects models were constructed to identify program factors associated with program selection.

**Results:**

Participants completed up to 8 discrete choice comparison exercises each resulting in 23,889 exercises were completed by 3054 participants. Selected programs were less likely to focus on prevention (vs. treatment). For each 1-year increase in the marginal years of life gained, there was a 15% increase in the odds of a program being selected. Interventions tailored to marginalized communities or targeting stigmatized health conditions were less likely to be selected compared to interventions targeted to the general population or targeting chronic health conditions. Noteworthy exceptions included an increased preference for interventions aligning with the perceived needs or cultural expectations for marginalized communities.

**Conclusions:**

Stigmatizing perceptions of health conditions and key populations likely influence public health programming preferences of Canadians.

**Public health implications:**

Informational campaigns highlighting disparities experienced by marginalized populations may improve support for targeted and tailored interventions.

## Introduction

The rise of statistical thinking and evidence-based decision making has promoted the idea that public health decisions are shaped primarily by scientific findings [1, 2]. In reality, public health systems are socially constructed – meaning that while scientific findings may play an important role in decision making, the extent of their influence is mediated by the social environments within which they exist [3–6]. In Canada, most public health activities are influenced by federal parliament, provincial legislative assemblies, ministerial bureaucracies, professional unions and colleges, healthcare organizations, and, of course, patients and providers themselves [1, 7]. Public health is therefore vulnerable to and often serves to reproduce the biases of these individuals and institutions [8–10].

Understanding how bias operates within these social environments is important for improving decisions regarding how policies are made, priorities are set, and resources are allocated. These decisions, after all, are the basis of public health programming: they determine what inputs and resources are mobilized, what activities are undertaken, and thereby shape program outputs and outcomes. In short, such decisions determine *what* harms are addressed, *how*, and for *whom* [11]. The factors that enter into these decisions are diverse and manifold. They may include considerations of which conditions are most harmful or urgent to address, which are treatable or preventable, which populations are most greatly impacted, and the relative opportunity costs of addressing one problem over another. While these factors are undoubtedly worthy of consideration, the fact that they are socially constructed means they are vulnerable to bias [12–14].

It is increasingly recognized that biased policy decisions tend to disadvantage and harm marginalized communities [15]. To prevent these harms, equity oriented frameworks have been used to promote tailored and targeted public health interventions that aim to directly promote health and wellness among marginalized communities [16–19]. Yet, the implementation of these frameworks and programs requires the support of policy-makers and the institutions that empower them [20]. Thus, the very social conditions that have given rise to health disparities must be engaged in order to address them. This poses a considerable challenge to equity-oriented public health programs in many jurisdictions – particularly when marginalized communities continue to face stigma and discrimination.

Understanding how the public conceptualizes the need for tailored and targeted public health programs may help to address barriers to health equity. While the public’s influence on health policy decisions is mediated through institutional processes, we nonetheless conceptualize public opinion as relatively important – especially when analyzing public health systems of developed democracies. This position is supported by previous work which suggests that public perceptions and support of health and social programs may play an important role in facilitating policy improvements [21]. Previous research has examined the preferences of the public and policy makers, but these have usually focused on age, disease severity, and income, with little attention to specific marginalized identities [22–26]. To contribute to our understanding of public views regarding tailored and targeted public health programs for specific marginalized groups, we conducted a discrete choice experiment that elicited resource allocation preferences of Canadians. We selected a discrete choice experiment design because it allowed us to estimate the preferences of survey participants by the choices they made in head-to-head comparisons between competing hypothetical programs [27–29]. This design further allowed us to compare trade-offs in people’s preferences, which provides insight into the potential weight that various factors might play in influencing whether participants prefer one program over another [27–29]. For example, we can understand the trade off between years of life gained and the population of interest to understand how participants might value equity-specific gains compared to total population gains. In undertaking this discrete choice study, we hypothesized that individuals would favor programs designed for the general population, as opposed to those designed for marginalized communities or stigmatized health conditions.

In recognition of their ability to efficiently identify preferences in healthcare delivery and policy [30, 31], discrete choice experiments have been increasingly utilized and applied in healthcare and health policy research [27–29]. The present study was particularly influenced by Lal et al. (2019), which used a discrete choice experiment to examine whether participants preferred health programs designed for low vs. high income groups [32]. Their results showed that the preference weight attributable to income was similar to the estimated weight of epidemiological disease burden for low vs. high income groups. In another influential study, Luyten et al. (2015) used a discrete choice experiment to demonstrate that patient life-style and age were important determinants of whether survey participants preferred a given intervention [33]. The present study builds off these previous studies to explicitly examine preferences related to treatment and prevention across a range of health conditions, for a diverse set of population groups.

## Methods

### Participant recruitment

Participants for our study were recruited online using paid advertising on Facebook and Instagram between May 13th, 2020 and June 15th, 2020, during which time Canadians were exiting the third wave of the COVID-19 pandemic and seeing breaking national headlines about mass burial sites at Indigenous residential schools [34, 35] (both of which are factors that we acknowledge may have considerable potential to influence perceptions on healthcare programming). Recruitment preceded for 1 month to support engagement of infrequent social media users. After clicking on a paid advertisement in either English or French, participants were screened for eligibility. Eligibility criteria restricted participation to individuals who (1) were 16 years of age or older, (2) reported living in Canada, (3) provided informed consent, and (4) were able to complete the questionnaire in English or French.

### Data collection

**Fig. 1:**
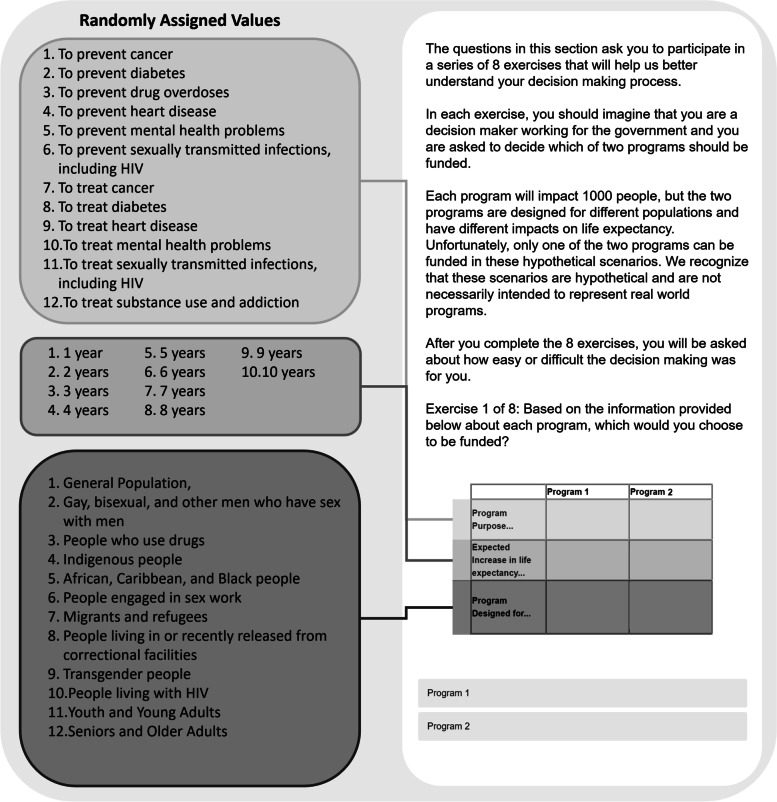
Design of Discrete Choice Experiment

The survey questionnaire was hosted on the Qualtrics Platform. After being screened for eligibility and providing informed consent, participants participated in a discrete choice experiment (See Fig. 1). The experiment consisted of 8 exercises in which participants were asked to imagine that they were a decision maker choosing which of two public health programs should be funded. Each program was described by its (1) purpose, (2) expected increase in life expectancy, and its (3) target audience. JavaScript was used to randomly generate the descriptors for each program from a list of 12 program purposes (i.e., *To prevent cancer; To prevent diabetes; To prevent drug overdoses; To prevent heart disease; To prevent mental health problems; To prevent sexually transmitted infections, including HIV; To treat cancer; To treat diabetes; To treat heart disease; To treat mental health problems; To treat sexually transmitted infections, including HIV; To treat substance use and addiction*), 10 life expectancy estimates (i.e., *1–10 years*), and 12 target populations (i.e., *General Population; Gay, bisexual, and other men who have sex with men; People who use drugs; Indigenous people; African, Caribbean, and Black people; People engaged in sex work; Migrants and refugees; People living in or recently released from correctional facilities; Transgender people; People living with HIV; Youth and Young Adults; Seniors and Older Adults*). The study was piloted with a group of 10 undergraduate and graduate students and a pilot study with 52 responses was used to identify issues or participant concerns/complaints prior to launching the study. Slight revisions were made based on these data and thus they were not included in the present study.

### Data analysis

To adjust for sampling biases, we conducted iterative proportional fitting using the *anesrake* package in R (v. 4.0.3). Iterative proportional fitting is a statistical weighting approach that allows for the construction of a single weight based on several participant characteristics [36]. It is advantageous when the population distribution of each weighted variable is known, but when the intersections of these weights are uncertain [37]. The raking algorithm used to iteratively fit the population proportion assigns weights repeatedly to achieve convergence until all target variables are within 5% of the target weights. Using iterative proportional fitting allowed us to generate statistical weights that could be used in our descriptive and multivariable analyses. Target weights, reported in Table 1, were based off the Canadian Census Population (adjusting for age, gender, ethnicity, educational attainment, income, province of residence). As the data were collected via an online, opt-in survey, we also adjusted for the average time participants spent using social media (based on estimates from the Ryerson Social media Lab) [38] and political orientation (based on estimates from CBC News Poll Tracker Averages) [39]. Descriptive statistics were used to provide an overall description of the sample and assess how frequently programs with each characteristics were selected when participants were presented with an opportunity to choose programs with each given characteristic. A weighted generalized linear mixed-effects model was constructed using the *lme4* package to identify program factors associated with program selection preferences. The outcome factor indicated whether the program was selected. A random effect term was used to account for within-person effects arising from the repeated observations (i.e., up to 8 per participant). Explanatory factors included measures of the program purpose, the target population, whether the intervention focused on a prevention or treatment approach, and the relative years of life gained for the selective (vs. unselected) program. An interaction term between the program purpose and target audience was included. Person-level characteristics were not analyzed in our multivariable model as all program characteristics were randomized to participants. Categorical data are regressed in the *lme4* using the contrast schemes (also called dummy coding). As dummy variable coding yields the same estimates for variable attribute differences as the alternative approach (i.e., effects coding), we opted to retain the contrasts scheme as it is widely used in the public health field, currently implement in R, and offered a relatively easy interpretation given our research questions [40, 41]. Given our use of the dummy coding scheme, readers should be cautious to interpret categorical variables relative to the selected reference-levels (which were selected based on interpretability and consistency in illustrating our hypotheses). Results are reported consistent with the ESTIMATE checklist, which represents the ISPOR guidelines and recommendations for the conduction of discrete choice experiments [42, 43].

**Table 1:** Participant Characteristics

	Unweighted	Target	Weighted
	N (%)	(%)	N (%)
**Age**			
16 to 30 years	165 (5.4)	22.6	552.8 (18.1)
31 to 64 years	1654 (54.2)	57.9	1823.1 (59.7)
65 years or older	1235 (40.4)	19.5	678.1 (22.2)
**Gender**			
Man	1227 (40.2)	49.3	1465.0 (48.0)
Woman	1569 (51.4)	50.6	1584.7 (51.9)
Non-binary	105 (3.5)	0.1	4.3 (0.1)
**Province**			
Atlantic Canada	264 (8.6)	6.6	220.7 (7.2)
British Columbia	720 (23.6)	13.2	417.4 (13.7)
Ontario	1018 (33.3)	38.3	1200.5 (39.3)
The Prairies	886 (29.0)	18.3	573.4 (18.8)
Quebec	151 (4.9)	23.2	632.0 (20.7)
The Territories	15 (0.5)	0.3	9.9 (0.3)
**Ethnicity**			
African, Caribbean, or Black	84 (2.8)	3.5	76.2 (2.5)
Indigenous	159 (5.2)	4.9	190.0 (6.2)
White	2237 (73.2)	72.8	2351.1 (77.0)
Other	574 (18.8)	18.8	436.7 (14.3)
**Educational Attainment**			
High School Diploma or Lower	471 (15.4)	44.7	1335.0 (43.8)
Advanced Training, below Bachelors level	1239 (40.6)	32.0	1008.4 (33.1)
Advanced Training, Bachelors level or Above	1342 (44.0)	23.3	705.6 (23.1)
**Income**			
Under $30,000	578 (18.9)	45.1	1343.7 (44.0)
$30,000 to less than $60,000	779 (25.5)	21.2	722.0 (23.6)
$60,000 to less than $90,000	653 (21.4)	23.3	333.6 (10.9)
$90,000 or more	1044 (34.2)	10.4	654.7 (21.4)
**Political Affiliation**			
Bloc Québécois	65 (2.1)	6.8	189.0 (6.2)
Conservative Party of Canada	1432 (46.9)	28.8	967.8 (31.7)
Green Party of Canada	294 (9.6)	4.6	234.6 (7.7)
Liberal Party of Canada	563 (18.4)	35.3	1087.7 (35.6)
New Democratic Party (NDP)	700 (22.9)	19.4	574.8 (18.8)
**Time Spent on Social Media**			
Less than 30 minutes per day	1677 (54.9)	50.0	1518.8 (49.7)
More than 30 minutes per day	1377 (45.0)	50.0	1535.1 (50.2)

## Results

Participants completed up to 8 discrete choice comparison exercises each resulting in 23,889 exercises were completed by 3054 participants. Table 1 shows the unweighted and weighted descriptive statistics of the sample, as well as the target prevalence used in the creation of statistical weights. In brief, youth and young adults, men, residents of Quebec, less educated individuals, and lower income individuals were under represented in the survey, as were those who identified with the Liberal and Green parties. Statistical weights were used to adjust for these biases, resulting in an analytic sample that closely represented the demographic characteristics of the Canadian population: 57.9% were between 31 and 64 years of age, 49.3% identified as male, 72.8% identified as white, 55.3% had received education or training beyond a high school diploma, and 54.9% had annual incomes greater than $30,000 CAD.

**Fig. 2:**
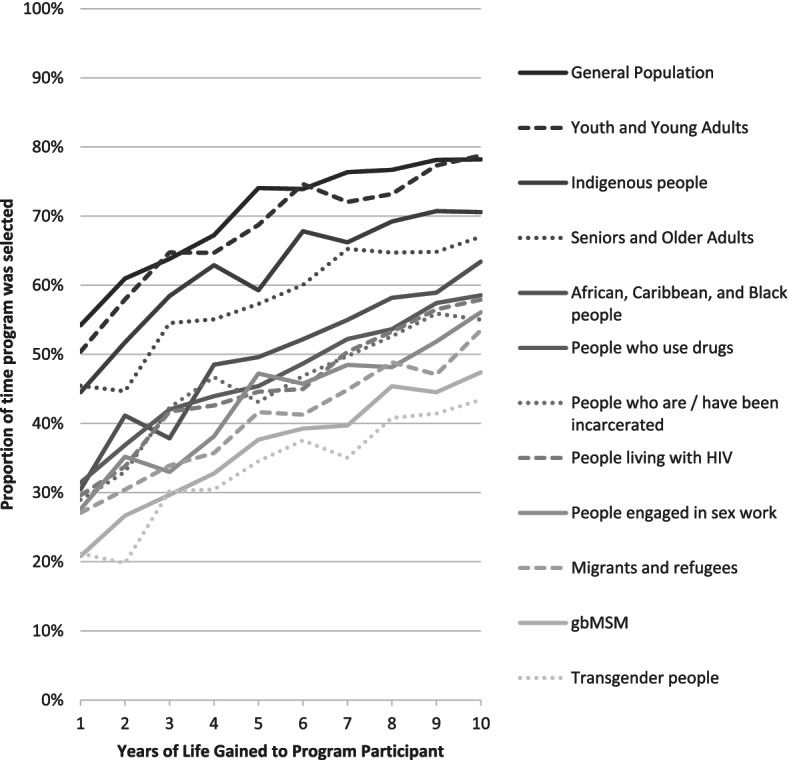
Proportion of time a program serving each client group was selected, by years of life gained to program participant

Table 2 shows the proportion of time interventions were selected that focused on each health condition and target audience. Programs targeting the general population were the most frequently selected (70.5%), followed by those support youth and young adults (68.3%), Indigenous peoples (62.1%), and seniors and older adults (57.8%). Interventions targeting migrants and refugees (40.3%), gay and bisexual men (36.1%) and transgender people (33.4%) were the least frequently selected. Figure 2 shows that as the number of life years gained for a given intervention increased, it was selected more frequently by participants. This was true for all sub populations reviewed, though we did not test for differences in slopes. Interventions for mental health (56.0%), cancer, (53.4%), diabetes (53.2%) and heart disease (51.6%) were all selected at least half of the time, while those targeting substance use (47.7%) and HIV or other sexually transmitted infections (43.5%) were selected slightly less than half of the time they appeared.

**Table 2:** Proportion of time a program serving each client group was selected when displayed, stratified by health condition

	% Population Selected, Overall	Mental Health	Cancer	Diabetes	Heart Disease	Substance Use	HIV & STIs
% Condition Selected, Overall	–	56.0	53.4	53.2	51.6	47.7	43.5
	%	%	%	%	%	%	%
**General Population**	70.5	75.8	70.6	75.1	76.8	68.2	62.1
**Youth and Young Adults**	68.3	77.2	70.8	71.9	65.7	67.3	61.9
**Indigenous people**	62.1	66.0	69.2	68.2	62.4	60.0	54.1
**Seniors and Older Adults**	57.8	68.5	60.6	67.4	64.5	54.6	40.4
**African, Caribbean, and Black people**	49.6	51.8	59.7	57.1	56.8	42.4	40.2
**People who use drugs**	47.0	56.8	46.7	44.9	42.9	51.2	40.0
**People who are/have been incarcerated**	45.5	57.8	42.3	45.8	41.6	47.0	39.6
**People living with HIV**	45.5	46.2	51.3	48.9	48.9	40.3	43.1
**People engaged in sex work**	43.1	47.1	38.9	43.2	39.7	44.7	42.3
**Migrants and refugees**	40.3	45.0	48.4	41.8	45.8	31.4	37.9
**gbMSM**	36.1	38.7	40.7	35.7	38.9	33.9	33.0
**Transgender people**	33.4	42.9	39.3	36.5	34.9	28.2	26.3

Results of multivariable analyses are shown in Table 3. Selected programs were less likely to focus on prevention (vs. treatment; aOR = 0.87, 95% CI = 0.84–0.91). For each 1-year increase in the marginal years of life gained, there was a 15% increase in the odds of a program being selected (Adjusted Odds Ratios [aOR] = 1.15, 95% Confidence Intervals [CI] = 1.14–1.15). Interventions targeting African, Caribbean, and Black people (aOR = 0.56, 95% CI = 0.41–0.77); Gay, Bisexual, and other Men Who Have Sex with Men (gbMSM; aOR = 0.24, 95% CI = 0.17–0.33)); Migrants and refugees (aOR = 0.26, 95% CI = 0.20–0.36); People engaged in sex (aOR = 0.24, 95% CI = 0.18–0.33); people who are/have been incarcerated (aOR = 0.19, 95% CI = 0.14–0.26); people living with HIV (aOR = 0.52, 95% CI = 0.37–0.72); people who use drugs (aOR = 0.34, 95% CI = 0.25–0.47); and transgender people (aOR = 0.26, 95% CI = 0.19–0.36) were less likely to be selected compared to interventions targeted to the general population.

**Table 3:** Factors associated with whether a program was selected (vs. not selected), with Client*Condition Interaction

	aOR	95% CI		aOR	95% CI		aOR	95% CI		aOR	95% CI		aOR	95% CI		aOR	95% CI	
**Treatment or Prevention** *(Ref: Treatment)*																		
Prevention	**0.87**	**0.84**	**0.91**	–	–	–	–	–	–	–	–	–	–	–	–	–	–	–
**Difference in Years of Life Gained** *(Per 1 year)*	**1.15**	**1.14**	**1.15**	–	–	–	–	–	–	–	–	–	–	–	–	–	–	–
**Client Population** *(Ref: General Population)*																		
African, Caribbean, and Black people	**0.56**	**0.41**	**0.77**	–	–	–	–	–	–	–	–	–	–	–	–	–	–	–
gbMSM	**0.24**	**0.17**	**0.33**	–	–	–	–	–	–	–	–	–	–	–	–	–	–	–
Indigenous people	0.91	0.67	1.25	–	–	–	–	–	–	–	–	–	–	–	–	–	–	–
Migrants and refugees	**0.26**	**0.20**	**0.36**	–	–	–	–	–	–	–	–	–	–	–	–	–	–	–
People engaged in sex work	**0.24**	**0.18**	**0.33**	–	–	–	–	–	–	–	–	–	–	–	–	–	–	–
People who are/have been incarcerated	**0.19**	**0.14**	**0.26**	–	–	–	–	–	–	–	–	–	–	–	–	–	–	–
People living with HIV	**0.52**	**0.37**	**0.72**	–	–	–	–	–	–	–	–	–	–	–	–	–	–	–
People who use drugs	**0.34**	**0.25**	**0.47**	–	–	–	–	–	–	–	–	–	–	–	–	–	–	–
Seniors and Older Adults	1.10	0.79	1.51	–	–	–	–	–	–	–	–	–	–	–	–	–	–	–
Transgender people	**0.26**	**0.19**	**0.36**	–	–	–	–	–	–	–	–	–	–	–	–	–	–	–
Youth and Young Adults	1.22	0.88	1.70	–	–	–	–	–	–	–	–	–	–	–	–	–	–	–
**Health Condition** *(Ref: Cancer)*																		
Diabetes	**1.56**	**1.15**	**2.11**	–	–	–	–	–	–	–	–	–	–	–	–	–	–	–
Heart disease	**1.75**	**1.30**	**2.34**	–	–	–	–	–	–	–	–	–	–	–	–	–	–	–
HIV/STIs	**0.60**	**0.46**	**0.78**	–	–	–	–	–	–	–	–	–	–	–	–	–	–	–
Mental health problems	1.08	0.82	1.43	–	–	–	–	–	–	–	–	–	–	–	–	–	–	–
Substance use	0.91	0.70	1.19	–	–	–	–	–	–	–	–	–	–	–	–	–	–	–
Client*Condition *(Ref: General Population)*	**Cancer**			**Diabetes**			**Heart Disease**			**HIV/STIs**			**Mental Health**			**Substance Use**		
African, Caribbean, and Black people	1.00			**0.48**	**0.32**	**0.73**	**0.53**	**0.35**	**0.79**	0.76	0.52	1.10	0.69	0.47	1.03	**0.60**	**0.41**	**0.87**
gbMSM	1.00			**0.47**	**0.31**	**0.71**	**0.55**	**0.37**	**0.83**	**1.73**	**1.18**	**2.53**	0.89	0.60	1.33	1.00	0.68	1.47
Indigenous people	1.00			**0.55**	**0.36**	**0.82**	**0.32**	**0.21**	**0.47**	0.83	0.58	1.19	**0.62**	**0.42**	**0.93**	0.75	0.52	1.09
Migrants and refugees	1.00			**0.50**	**0.33**	**0.74**	**0.61**	**0.41**	**0.90**	**1.91**	**1.33**	**2.74**	1.15	0.78	1.68	0.76	0.53	1.10
People engaged in sex work	1.00			**0.56**	**0.37**	**0.85**	**0.52**	**0.35**	**0.78**	**1.78**	**1.23**	**2.57**	1.07	0.72	1.60	1.18	0.81	1.72
People who are/have been incarcerated	1.00			0.86	0.57	1.29	0.72	0.48	1.06	**2.20**	**1.53**	**3.16**	**2.41**	**1.64**	**3.56**	**1.92**	**1.33**	**2.77**
People living with HIV	1.00			**0.39**	**0.25**	**0.59**	**0.45**	**0.30**	**0.68**	0.99	0.68	1.45	**0.61**	**0.40**	**0.91**	**0.58**	**0.40**	**0.86**
People who use drugs	1.00			**0.57**	**0.38**	**0.86**	**0.37**	**0.25**	**0.55**	1.13	0.78	1.64	**1.53**	**1.03**	**2.28**	**1.45**	**1.00**	**2.11**
Seniors and Older Adults	1.00			**0.40**	**0.27**	**0.62**	**0.36**	**0.24**	**0.54**	**0.35**	**0.24**	**0.50**	**0.61**	**0.41**	**0.92**	**0.44**	**0.30**	**0.65**
Transgender people	1.00			**0.55**	**0.37**	**0.84**	**0.48**	**0.32**	**0.73**	0.98	0.68	1.43	1.11	0.75	1.66	0.70	0.48	1.04
Youth and Young Adults	1.00			**0.55**	**0.35**	**0.85**	**0.31**	**0.20**	**0.48**	0.79	0.54	1.15	0.98	0.64	1.49	0.93	0.63	1.38

Relative to interventions addressing cancer, those addressing diabetes (aOR = 1.56, 95% CI = 1.15–2.11) and heart disease (aOR = 1.75, 95% CI = 1.30–2.34) interventions were preferred, while those addressing HIV and other STI’s had lower odds of being preferred (aOR = 0.60, 95% CI = 0.46–0.78). The Client-Condition interaction term revealed notable exceptions to these patterns: HIV & STI interventions for gbMSM (aOR = 1.73, 95% CI = 1.18–2.53), migrants and refugees (aOR = 1.91, 95% CI = 1.33–2.74), people engaged in sex work, (aOR = 1.78, 95% CI = 1.23–2.57), and people who are or have been incarcerated (aOR = 2.20, 95% CI = 1.53–3.16) were more likely to be selected and those HIV & STI interventions targeting seniors and older adults were less likely to be selected (aOR = 0.35, 95% CI = 0.24–0.50). Mental health interventions targeting Indigenous people (aOR = 0.62, 95% CI = 0.42–0.93), people living with HIV (aOR = 0.61, 95% CI = 0.40–0.91), and seniors/older adults (aOR = 0.61, 95% CI = 0.41–0.92) were less likely to be selected while those targeting people who are or have been incarcerated (aOR = 2.41, 95% CI = 1.64–3.56) or who used drugs (aOR = 1.53, 95% CI = 1.03–2.28) were more likely to be selected. Similarly, substance use interventions targeted to people who are or have been incarcerated (aOR = 1.92, 95% CI = 1.33–2.77) or people who used drugs (aOR = 1.45, 95% CI = 1.00–2.11) were more likely to be selected, while those targeted to African, Caribbean, and Black people (aOR = 0.60, 95% CI = 0.41–0.87), people living with HIV (aOR = 0.58, 95% CI = 0.40–0.86), and seniors/older adults (aOR = 0.44, 95% CI = 0.30–0.65) were less likely to be selected. Finally, interventions addressing diabetes or heart disease among all populations (except people who were or had been incarcerated) were less likely to be selected (See Table 3 for aORs and 95% CIs).

## Discussion

### Primary findings

We conducted a discrete choice experiment asking participants to select between competing public health programs and aimed to identify program characteristics associated with the selected programs. Supporting previous findings and current practice in cost-benefit analyses [22, 23, 44], the results of our discrete choice experiment showed that participants were more likely to select interventions that contributed to greater gains in life expectancy [24, 26, 45]. We also observed that participants were more likely to select programs focused on treatment as opposed to prevention. The existing literature on this issue has been mixed, with data from the Swiss Household Panel Survey suggesting that the majority of citizens support preventative interventions over treatment-based ones [46] while other discrete choice experiments have shown a preference for treatments of more severe illness over less severe illness [33]. Our findings support the latter, showing that participants prioritize treatment over prevention.

Extending the existing literature, our study also examined the role of specific health conditions and target populations in shaping funding preferences. In doing so, we found that participants were less likely to select interventions targeting stigmatized health conditions (e.g., HIV and Other STIs) and more likely to select interventions targeting relatively more common chronic health conditions, such as heart disease and diabetes. This conforms with other research that highlight the role of stigma in shaping attitudes towards specific health conditions [47–49]. Similarly, participants were less likely to select interventions tailored to key populations, favoring instead those that were tailored to the general population. These findings align with previous research by Skedgel, Wailoo, and Akehurst (2015) which showed that participants generally support interventions that maximize life expectancy gains, but that they are willing to prioritize smaller gains to *preferred groups* over larger gains to *less preferred* groups [24]. These findings therefore highlight potential mechanisms in which marginalized groups are disadvantaged by public opinion. Likewise, Norman et al. (2013) showed that participants tend to favor programs for individuals like themselves, which given our sample would contribute to the less support for marginalized individuals [50]. Optimistically, the present study highlights several notable exceptions to this general pattern: Participants were more likely to select interventions tailored to populations widely known or believed to be affected by specific health conditions. For example, interventions addressing HIV and other STIs were more likely to be selected when they were targeted to gbMSM, migrants and refugees, people engaged in sex work, or people who are or have been incarcerated but were less likely to be selected when tailored to seniors and older adults. This finding aligns with research by Purtle (2020) showing that the public generally believes that evidence should have “a lot of influence” on health policy decisions [51]. Likewise, these findings conform to work by Shmueli et al. (2017) and Olsen & Richardson (2013) which demonstrate the public’s support for equity and equality in healthcare priorities [52, 53]. Importantly, we note that participants support for these programs may arise from both accurate (i.e., awareness of epidemiological burden) or inaccurate (i.e., cultural assumptions about the “riskiness” of marginalized groups) perceptions of the need for care within these populations. Certainly, gbMSM and other key populations do experience a disproportionate burden of disease from HIV and other STIs, but these burdens are experienced regardless of whether they are recognized by the public. For example, substance use interventions targeted to Indigenous people were not more likely to be selected in our study, despite the fact that tailored and targeted programs are needed to support Indigenous people who use drugs [54–56]. Likewise, tailored and targeted interventions addressing mental health and substance use are needed for gbMSM, even though this need may not be as widely recognized as their need for interventions addressing HIV and other STIs [57]. These findings suggest that the public may not be sufficiently knowledgeable about the inequities facing key populations and that if they were, they might be more supportive of tailored and targeted interventions for high-need populations.

### Limitations

The present study has limitations. First, this study leveraged data from a web-based survey advertised on social media platforms resulting in the under-representation of several key populations. While iterative proportional fitting was used to address this bias, it is impossible to know the extent to which hidden factors may have influenced recruitment of participants. Second, our discrete choice experiment was implemented without design restrictions – meaning that some of the combinations of life expectancy gains, target populations, and program areas are unlikely to be advanced or achieved. Our choice to keep all intersections was informed both by our technical capacity in implementing the study via the Qualtrics platform, as well as our desire to understand the full spectrum of effects. For example, comparing less commonly implemented interventions (e.g., Cardiovascular disease interventions for gay and bisexual men) helps us to understand when and when not the public might be supportive of an intervention (e.g., HIV/STBBI interventions for gay and bisexual men). Third, the present study does not allow us to understand how and why participants made the choices they did. Additional qualitative research or more refined quantitative methods are needed to explore the ways in which individuals think about the choices they made. This work is important to confirm our argument that participant’s preferences for some population-specific programming arises from the populations awareness or perceptions of needs for these marginalized groups. We acknowledge, however, that stigma and other factors could also play a role in shaping preferences. Fourth, we note that we did not conduct a formal power analyses for this study. Rather the number of participants recruited was based on budgetary restraints. Information on power calculation for Discrete Choice Experiments is available from Bekker-Grob (2015) [58]. In the present study, each intersection of target population and condition was presented approximately 330 times. Further work is needed to assess the relative contributions of these various factors.

### Future research

As noted above, preferences for resource allocations are likely informed by participant’s characteristics. While we have controlled for participant demographic characteristics in the present analyses, the effect that participant identity on participant preferences was not explored. These analyses are feasible in the present dataset. However, we feel that a more nuanced, dedicated, and focused introduction and discussion are needed to contextualize the role of identity in shaping stated preferences as well as an exploration of potential mechanisms that might influence whether an individual is likely to support an intervention tailored for marginalized peoples. Such work will be explored in future analyses.

## Conclusion

The findings of the present study suggest that the public generally underestimates the need for tailored and targeted public health programming and may undervalue the benefits of preventive public health efforts relative to treatment-based programs. These results highlight the potential utility of educating the public about health inequities experienced by key populations. We found that while participants generally favored interventions designed for the general public, they were willing to support tailored and targeted interventions in select cases: likely those that conformed with their perceptions of need within key populations. Of course, caution should be taken to ensure that health disparities are not inappropriately attributed to the identities of marginalized and oppressed individuals, but rather the broader social and structural determinants that give rise to inequities.

## Data Availability

The datasets used and/or analysed during the current study are available from the corresponding author on reasonable request.
